# Principal component analysis suggests multiple dimensions of memory inhibition that are differentially affected by age

**DOI:** 10.3389/fpsyg.2022.1020915

**Published:** 2023-01-30

**Authors:** Fabian W. Corlier, Teal S. Eich

**Affiliations:** Leonard Davis School of Gerontology, University of Southern California, Los Angeles, CA, United States

**Keywords:** memory, inhibition, aging, cognition, PCA

## Abstract

**Background:**

Cognitive inhibition is among the executive functions that decline early in the course of normal aging. Failures to be able to inhibit irrelevant information from memory may represent an essential factor of age-associated memory impairment. While a variety of elaborate behavioral tasks have been developed that presumably all index memory inhibition, the extent to which these different tasks measure the same underlying cognitive construct that declines with age has not been well explored.

**Methods:**

In the current study, 100 and 75 cognitively healthy younger (*n* = 71; age = 30.7 ± 5.4 years, 56.7% female) and older (*n* = 104, age = 69.3 ± 5.9 years, 66.2% female) adults with equivalent educational attainment performed three computer-based memory inhibition tasks: the Retrieval Induced Forgetting task, the Suppress task, and the Directed Forgetting task. We conducted a principal component analysis using scores derived from different components of these tasks to explore whether and how the tasks relate to one another. We further investigated how age, sex and education, along with, in a subsample of the participants, a neuropsychological measure of episodic memory, impacted both the task scores individually, and the principal components derived from the exploratory analysis.

**Results:**

We identified 3 distinct sources of variability which represent potentially independent cognitive processes: memory retrieval facilitation, and two memory inhibition processes that distinguished themselves by the degree of volitional initiation of memory suppression. Only the memory retrieval component correlated with a neuropsychologically-derived episodic memory score, and both memory inhibition principal components were age dependent.

**Conclusion:**

Our findings provide support for a distinction in memory suppression processes between those ‘instructed’ to be performed and those which happen without explicit instruction. This distinction adds nuance to the dichotomous classification of controlled vs. automatic inhibitory mechanisms, which have been shown in previous work to vary as a function of the degree of frontal involvement. Our findings further demonstrate that while both of these measures of inhibition were affected by age, the episodic memory component was not, suggesting that inhibitory impairments may precede memory deficits in healthy aging.

## Introduction

It is well established that cognitive functioning declines across multiple cognitive domains, even during healthy (non-pathological) aging (Bayles and Kaszniak, 1987; Craik and Salthouse, 2011). The most prevalent complaint among older adults is a failure to be able to remember (Schacter, 1999). According to multiple lines of evidence, two distinct memory systems are implicated in such declines (Gabrieli, 1996; Wang and Snyder, 1998; Buckner, 2004). The first system includes the medial temporal formation which is among the first to be impacted in late onset Alzheimer’s disease and is typically associated with episodic memory. The second system includes executive systems that support memory with a dominantly frontal involvement (Buckner, 2004). In support of the latter, many studies suggest that declines in processing speed, attentional resources and executive functions are responsible for the memory impairment in older adults (Mcdowd and Craik, 1988; Salthouse, 1996; Tsang, 1998; Verhaeghen and Cerella, 2002; Craik and Salthouse, 2011). The underlying mechanisms that explain this decline, however, are less clear. As aptly stated by Glisky (2007): “slowed processing, like attentional resources, is more a descriptor of aging cognition than an explanation for cognitive deficits and says nothing about what causes slowing with age.”

In a pivotal article, Hasher and Zacks (1988) argued that the notion of cognitive resources may represent different cognitive processes, depending on the demands of the task under consideration. They proposed, instead, that inhibitory control plays a fundamental role in many age-related cognitive deficits, and described how an inhibitory account more parsimoniously explains experimental evidence than does the resources theory (Hasher and Zacks, 1988; Hasher et al., 2007). In particular, older adults’ higher vulnerability to sensory distractions—for instance, noises or visual clues when performing a task—demonstrates that they have spared excitatory attentional resources but lack the inhibitory capacity to filter out these distractors from focal attention, which leads to increased interference and memory failures.

A large body of literature corroborates the idea that inhibitory processes, or those processes that allow an individual to select for or against information, worsen with age (Sylvain-Roy et al., 2015). However, the precise nature of inhibitory control remains unresolved. For example, it was initially hypothesized (e.g., Birren, 1959; Harnishfeger, 1995) that inhibition represented one cognitive process. Later work, however, demonstrated that there may be specific types of inhibitory processes (McDowd et al., 1995; Wilson and Kipp, 1998; Nigg, 2000; Friedman and Miyake, 2004), which act at different time points and on different mental representations. The most widely studied type is response inhibition, which is primarily concerned with the stopping of a prepotent motor action and is often assessed using a variety of different tasks including the stop signal reaction time task, the go/no go task, and the antisaccade task. While some studies have reported significant inter-correlations among these tasks (Friedman and Miyake, 2004), others have failed to confirm a model in which these commonly used tasks load together onto one factor (Gärtner and Strobel, 2021). Even within a given task, it is possible that the specific demands may also reveal distinct underlying mechanisms. A case in point is a study by Shilling et al. (2002) which showed that different variants of the same inhibitory task (the stroop) produced largely uncorrelated results.

Another proposed type of inhibitory control, which is the focus of the current report, surrounds information already stored in memory. Being able to effectively inhibit items from memory has been proposed to play a central role in successful long term (Anderson and Bell, 2001) and working (Blair, 2012) memory. When a medication dose is changed, for example, the old dose needs to be inhibited, lest it interferes with the new dose. In this way, reductions in the ability to inhibitory past memories may have direct implications for older adults’ ability to function independently in daily life, a factor in considering institutional care (Luppa et al., 2012).

As stressed by McDowd et al. (1995), the tasks used to investigate inhibitory processes must be carefully explained and analyzed to make *a priori* hypotheses about the expected performance while also recognizing and accounting for other age-related phenomena that could affect task performance. While numerous studies demonstrate that older adults have deficits in memorial suppression (see Lustig et al., 2007; Campbell et al., 2020), it is possible for older adults to bring inhibitory processes online under the right conditions. A study by Murray et al. (2015), for example, showed that providing a strategy seems to mitigate age-related inhibitory deficits. In their study, older adults were less able to successfully inhibit to-be-forgotten items in a think/no think task when they were provided with a simple, non-specific instruction for how to treat the to-be-forgotten items (e.g., just “keep the target word from coming to mind”). Yet, age-related deficits were ameliorated when more detailed, specific instructions were provided (e.g., participants “were to clear their mind entirely of the associated target word and focus their full attention on the cue word for the entire time it was on the screen. They were instructed *not* to think about other potential associates for the cue word, to play word games with the cue word, to repeat the cue word over and over in their mind, to shift their eyes away from the word or from the screen, or to do anything else that would distract them from thinking about the cue word”). These results point to a relatively easy to implement potential remediation technique to counter the widely reported deficits in older adults’ memory suppression abilities (c.f. Bulevich et al., 2006), though Murray et al. (2015) acknowledge that, in the wild, older adults may not engage in such active and adaptive strategies on their own.

Thus, both differences across tasks that are commonly used to study inhibition, as well as variations within the same task may lead to subtle, but important differences in how and what type of inhibitory control is carried out. In the present study, we sought to test the whether there exist multiple component processes within tasks commonly used to assess memory inhibition (Noreen and Mac Leod, 2015), and to probe whether and how these differences relate to sociodemographic factors including age, sex and education level in a naturalistic, (e.g., unguided) setting. To this end, we tested clinically healthy older and younger participants on three computerized tasks that have each been associated with memory inhibition in past research: an item-method Directed Forgetting task (Bjork and Woodward, 1973; Murayama et al., 2014), the Suppress task (Nee and Jonides, 2008), and a Retrieval Induced Forgetting task (Bjork, 1989; Anderson et al., 1994).

The first task was the item-method Directed Forgetting (DF) task (Muther, 1965). In this task, participants are provided with items one at a time and told, after the presentation of each item, whether the item should be remembered or forgotten for a later memory test. Memory for all items, including the items that should have been forgotten along with novel items, is then probed. The item method DF task, rather than the list method DF task, was chosen because it typically produces both larger and more reliable results (MacLeod, 1999; Sego et al., 2006; Zellner and Bauml, 2006). To account for differences in baseline episodic memory ability, a difference score between accuracy on the to-be-remembered and to-be-forgotten items, called the directed forgetting effect, is usually calculated. Younger adults typically show poorer memory for to-be-forgotten (TBF) items relative to to-be-remembered (TBR) items. A recent study by Fellner et al. (2020) modeled the time-frequency of EEG data to directly test whether this reduced memory for TBF items resulted merely from the passive decay of these items (together with a simultaneous enhancement of encoding of to-be-remembered items; see Bjork, 1972; MacLeod, 1999), or instead if it stemmed from an active inhibitory mechanism which left these items inaccessible at retrieval. Evidence largely supported the latter, with active downregulation of the TBF items resulting in forgetting (p. 2638). These findings are consistent with an earlier intercranial electrophysiological study by the same group, which also found evidence for an active, prefrontal-hippocampal inhibitory circuit whereby hippocampal encoding was inhibited by upstream signals originating in the dorsolateral prefrontal cortex, which led to forgetting (Oehrn et al., 2018). Whereas the directed forgetting effect is robust in younger adults (c.f. Gao et al., 2016, 2019; Zwissler et al., 2015), a 2010 meta-analysis by Titz and Verhaeghen (2010) revealed that this effect, while present, is “reliably smaller in older adults than in younger adults, even after controlling for age differences in baseline recall.” Interestingly, our group recently reported, based on a subset of the present data for which structural neuroimaging data was available, that cortical thickness in the inferior frontal gyrus partially explained these age-related differences in directed forgetting (Eich et al., 2021).

The second task, the Suppress task (Nee and Jonides, 2008), is modeled after the letter Sternberg task (Sternberg, 1966), in which a set of items is presented, and then memory for items that were or were not in the set is probed after a short delay. The Suppress task adds a key manipulation that allows for inhibitory ability to be investigated: following the word set, participants receive a cue which instructs them to keep in mind only half of the words (in this case, words of a specific color). After a delay, a memory probe consisting of one of the cued-color words (Valid), a novel (Control) word, or (unlike in the Sternberg) one of the non-cued color words (Lure) is tested. To the extent that the Lures are inhibited, familiarity for these items at test should be reduced. Thus, both the Lure probes, and the comparison of Control items (which should not be familiar because they were not present in the set) to the Lure items (the Lure-Control index) provide a measure of inhibitory ability. A smaller Lure-Control difference indicates better inhibitory ability, while a larger difference indicates a failure to appropriately drop the non-cued items from working memory. This task has been used to investigate memory inhibition across a number of different populations, including individuals with depression (Joormann et al., 2010), schizophrenia (Smith et al., 2011; Eich et al., 2014), and obsessive compulsive disorder (Ahmari et al., 2014). Our group has reported, using different variations of this task along with subsamples of the present data, that older adults have greater difficulty inhibiting irrelevant information from working memory relative to younger adults (Eich et al., 2018; Siegel and Eich, 2021), with the degree of memory inhibition deficit correlating with reduced cortical thickness in the left VLPFC (Eich et al., 2017).

The final task was the Retrieval Induced Forgetting task (for reviews see Anderson, 2003; Bäuml et al., 2010). In this multi-phase task, participants first incidentally encode a series of items, and then engage in cued-retrieval practice for a subset of these items, followed by a memory test for all items. The well-replicated finding is that the retrieval practice leads to the inhibition of related-but non-practiced items (Anderson et al., 1994). The prevailing theory surrounding the locus of this effect lies in an active inhibitory mechanism which acts to reduce the potential interference from competing items: memory traces for non-practiced but related items are suppressed to reduce their competition. The neural correlates of this task overlap to some extent with those implicated in the Directed Forgetting task (bilateral VLPFC, right DLPFC), but also include the ACC, which Anderson and Hulbert (2021) argue plays a central role in detecting and then resolving the conflict elicited by the related, but unpracticed competitors.

Interestingly, while age-related effects have been frequently reported in the Directed Forgetting and Suppress tasks, research on the Retrieval Induced Forgetting effect in healthy older adults (Aslan et al., 2007; Hogge et al., 2008; Collette et al., 2009; Gómez-Ariza et al., 2009; Aslan and Bäuml, 2012; Ortega et al., 2012) as well as in patients with Alzheimer’s disease (Moulin et al., 2002) has shown age-invariance, suggesting that the cognitive processes engaged are relatively preserved with age.

In the current study, older and younger participants completed each of these three memory imbibition tasks. Our primary objectives were to (1) explore the relationship of different measures from these tasks to each other; and to (2) determine if and how these identified constructs are impacted by age in the absence of clinical or neurological disease (e.g., changes to cognition arising from normal senescent change rather than neurodegeneration), self-reported sex, and participant’s level of education.

## Materials and methods

### Transparency and openness

De-identified data, along with the analytic code (in the R programming language) used in this study is available at https://osf.io/n3r8u/. Materials can be found in the Appendix. The study design, hypotheses and analytic plan were not preregistered.

### Participants

A total of two hundred and five participants between the age of 20 and 40 or over 60 were recruited to the present study, called the SOFIA study (Study of the Factor-structure of Inhibition in Aging), which focused on inhibitory control in aging. SOFIA study participants had previously taken part in one of two larger studies in the Cognitive Neuroscience Division at the Columbia University Medical Center (CUMC), either the Reference Ability Neural Network study (RANN) or the Cognitive Reserve study (CR), and were recruited to these studies *via* random-market-mailing targeting individuals living within 10 miles of the CUMC. All participants were required to be native English speakers, strongly right-handed, free of MRI contraindications, hearing or visual impairment that would impede testing, and free of medical or psychiatric conditions that could affect cognition [as detailed in (Habeck et al., 2018)]. Older adult participants were additionally screened for dementia at their intake visit, and participants who met criteria were excluded. SOFIA study participants were recruited within 2 years of completing either a baseline or follow up visit in the RANN or CR study. SOFIA participants completed 10 different inhibitory tasks, including the three memory inhibition tasks that are the focus of the current report. The sample size for the SOFIA study was determined according to a power analysis for models considering all 10 of the administered tasks.

Prior to participating in the SOFIA study, informed consent, as approved by the Internal Review Board of the College of Physicians and Surgeons of Columbia University, was obtained for all participants. Participants were compensated for completing the study.

### Procedure

Participants were tested using Inquisit Millisecond software, and all testing occurred online. Testing took place across two testing sessions, each lasting approximately 1–2 h. At the beginning of each session, participants completed an instruction manipulation check (IMC) modeled after (Oppenheimer et al., 2009), in which they had to read a set of instructions and press an indicated key to advance to the next screen. The IMC helps identify impulsive behaviors or a possible lack of attention which could influence performance, and is useful for ensuring participant adherence/understanding of task instructions for online studies. We excluded 5 participants (2 younger and 3 older participants) with an IMC sum over the two sessions >6 and a difference between the two sessions <3. From the remaining 198 participants, 175 had complete data for all 3 memory inhibition tasks which are the focus of the current report.

### Experimental tasks

The behavioral data of interest included sub scores derived from three experimental tasks designed to measure memory inhibition, described below. The stimuli for all tasks were words, and no words were repeated across tasks.

#### Item-method directed forgetting task

**Figure 1:**
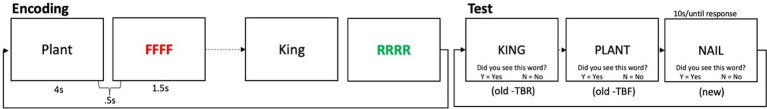
Schematic of the directed forgetting task. The 4 red Fs cued participants to forget the preceding word (e.g., Plant), whereas the 4 green Rs cued participants to remember the preceding word (e.g., “King”). Recognition memory for to-be-remembered (“TBR”), to-be-forgotten (“TBF”), and new (unpresented) words was then tested.

As is shown in Figure 1, the item method Directed Forgetting (DF) task had two phases: an encoding phase and a test phase. In the encoding phase, participants were presented with 36 words in the center of the screen, one at a time, for 2,500 ms each. Each word was followed 500 ms later by a cue: either four red letter Fs (for Forget) or four green letter Rs (for Remember), which remained on screen for 1,500 ms. The cue indicated whether the previous word was to-be-remembered (TBR) or to-be-forgotten (TBF) for a later memory test. The encoding phase consisted of 6 blocks of 6 trials, with equal numbers of TBR and TBF items in each block. The order of items within each block was randomized, and then fixed. In the recognition test phase, participants were presented, one at a time, with all 36 TBR and TBF words that they had encountered during the encoding phase, as well as 36 new words, in a blocked randomized order: 6 blocks of 12 words each were created by randomly taking 1 word from each study block, and ensuring that there were equal numbers of TBR and TBF words across each test block. The order of trials within each test block was then randomized, and fixed. Six additional filler words appeared as the first and last 3 items in the recognition phase, to control for primacy and recency effects, and were not analyzed. Before beginning the test, participants were told that they would be presented with words on the screen, and had to decide for each whether or not it had been presented in the earlier portion of the task. The instructions read: “If the word was presented before, press the Y key (for Yes). If the word was not presented before, press the N key (for No). NOTE: You should press the Y key for both words you were told to remember, and for words you were told to Forget, because all of these words were presented to you before.” Memory inhibition was operationalized as the difference in performance between TBR and TBF words, referred to as the Directed Forgetting (DF) score. To the extent that TBF words were inhibited, participants should indicate that these items were not presented (and rate them as new). Successfully inhibiting the TBF words, but correctly recognizing the TBR words, should therefore lead to a larger difference in performance. Thus, a larger DF score indicates better memory inhibition, whereas a smaller difference indicates a failure to appropriately suppress the to-be-forgotten items. We also considered accuracy on the TBR and TBF items individually.

#### Suppress task

**Figure 2:**
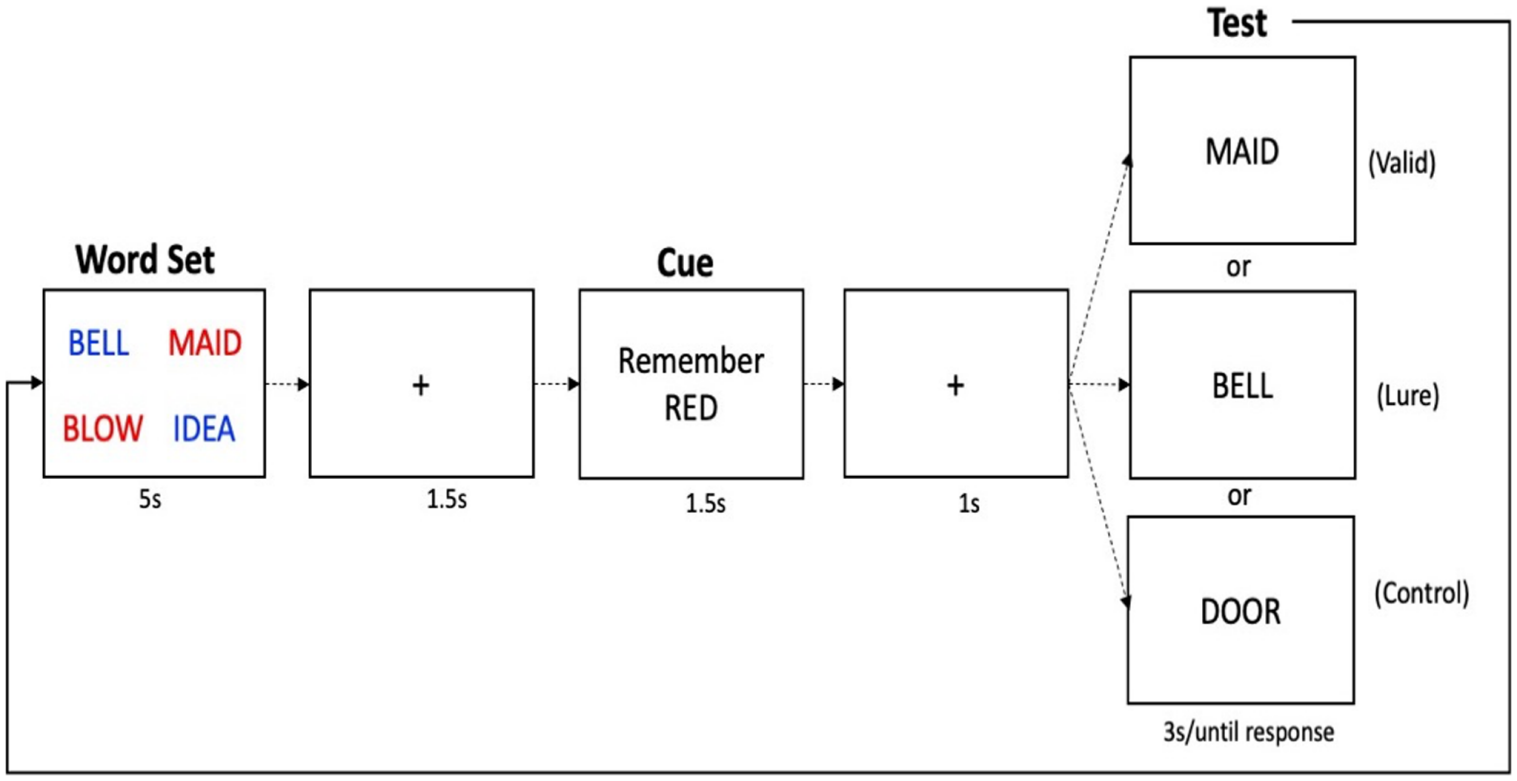
Schematic of the suppress task. One trial is shown, in which first the memory set is given, followed by a delay, a cue to remember either the red or blue words (red in this case), followed by a recognition probe, which asks “was this word one of the words you were supposed to remember?” Shown here are the three types of possible probes for each trial, although on each trial, only one probe is given.

An illustration of the Suppress task is shown in Figure 2. In this task, participants were first presented with four words, two printed in red, two printed in blue, in a 2 × 2 grid configuration, for 5 s. The word set was followed by a 1 s inter trial interval (ITI), and then a cue which told participants to either remember the red or the blue words. The cue remained on screen for 1.5 s. After another 1 s ITI, participants were presented with a test probe in the center of the screen. The test probe for each trial was one of three types: Valid (the test word was present in the array and was in the cued color); Lures (the test word was present in the array but was in the non-cued color); or Controls (the test word was not present in the array). Participants were told to press the “Y” key on the keyboard (for “Yes”) if the test probe was one of the words that they should still be holding in memory, or the “N” key (for “No”) if not. The test probe remained on screen for a maximum of 10 s. Of the test probes, 40% were Valid trials, which required a positive response, 30% were Lure trials in which required a negative response, and 30% were Controls, which also required a negative response. Participants completed four blocks of 25 trials. Before beginning the task, participants practiced until they achieved >60% accuracy. Feedback was not provided on experimental trials. Similar to the TBR words in the DF task which provide a baseline measure of long-term memory, the Valid trials in the Suppress task provide a baseline measure of working memory ability. Lure items, on the other hand, provide an index of inhibition: to the extent that Lure items are inhibited, they should not be familiar. Lure items that were not appropriately inhibited, on the other hand, will cause interference at the time of the probe, which will increase errors and reaction time for correct responses. The difference in performance between the Control probes and the Lure probes, called the “Suppress effect,” thus reflects inhibitory ability, where a larger difference indicates good inhibition, and a smaller (and more negative) difference indicates poorer inhibition. For accuracy, we inverted this score so that the direction of the RT and accuracy effects would be consistent. We also considered as variables the accuracy and latency scores for the Valid trials.

#### Retrieval induced forgetting

**Figure 3:**
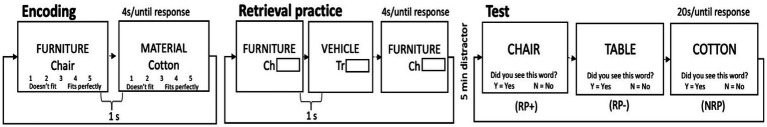
Schematic of the retrieval induced forgetting task. Shown here are two trials from the encoding phase, three trials from the retrieval practice phase, and three recognition probe trials. In this example, half of exemplars from the category ‘furniture’ were practiced, including ‘chair’ (and is hence an RP+ item), but not ‘table’, (and hence it is an RP-item). In this example, no items from the category ‘material’ received retrieval practice, and hence, all exemplars from this category (cotton, silk, burlap, etc…) are considered NRP items.

The third task, the Retrieval Induced Forgetting task (Bjork, 1989; Anderson et al., 1994), was composed of three phases: (1) an encoding phase, (2) a retrieval practice phase, and (3) a recognition test phase (see Figure 3). In the encoding phase, participants were presented with 6 blocks of 8 pairs of items. Each pair consisted of a category (e.g., FURNITURE), presented in capital letters, and an exemplar of the category (e.g., chair), presented below the category in lower case letters. Participants were given a maximum of 4 s to subjectively rate how well each exemplar represented the category. Each encoding trial was followed by a 1 s ITI. Following the encoding phase, participants completed retrieval practice on a subset of the items from a subset of studied categories. Participants were presented with 3 different blocks of 12 trials each (each repeated 3 times) which included half of the category-exemplar pairs from half of the categories presented during the encoding phase. In this phase, participants were given the category name as well as the first two letters of the target exemplar as retrieval cue, and had a maximum of 5 s to complete the word by typing the remaining letters (e.g., FURNITURE-ch___). A 1 s ITI separated each trial in this phase. Finally, after a 5-min distractor phase in which participants completed a visual go/no go task, participants completed the third and last phase, the recognition test. Participants were presented 6 blocks of 16 items, in a blocked-randomized design, and had to indicate whether the probe word had been presented during the encoding phase, or not. Participants had a maximum of 20 s to respond yes or no, by pressing either the Y or N key on the keyboard. The test phase interrogates the familiarity of items of three types: items that belonged to practiced categories whose exemplars received retrieval practice (RP+), items that belonged to practiced categories whose exemplars did not receive retrieval practice (RP-), and items whose categories received no retrieval practice (NRP). The first 3 blocks included only RP- items and half of the NRP items (called NRP-). The last 3 blocks included RP+ and the remaining NRP items (called NRP+, which were not analyzed). RP- items were always tested first to reduce the possibility of output interference due to the fact that RP+ items have higher strength in memory (Anderson et al., 1994). All blocks included 2 items from each of the 8 categories. The order of the stimuli for each test trial was determined randomly with the following constraints: the first item in the test block was a filler item (included to reduced primacy and recency effects, and not analyzed), and there were no immediate repeats of categories within or across blocks. The key variable of interest was the difference in performance between the RP- and NRP- items, of which there were equal numbers. A large literature has shown that the mnesic trace from unpracticed items that belong to practiced categories (i.e., RP- items) is weakened or extinguished by the practice of related category–exemplars, more so than for unpracticed items of unrelated categories (i.e., NRP items). Thus, the difference between accuracy for the RP- and NRP- items is our primary inhibitory variable, called the RIF effect. We also considered accuracy on RP+ items as an indicator of the effect of retrieval practice on memory retrieval facilitation.

### Episodic memory neuropsychological measure

A subset of 100 participants had previously completed the selective reminding test (SRT; Buschke, 1973) as part of the RANN or CR study. This pencil on paper neuropsychological test is frequently used to clinically assess episodic memory and is predictive of the development of dementia (Buschke and Fuld, 1974; Masur et al., 1990; Lemos et al., 2015). In this task, participants are presented with 12 unrelated words, and then must immediately recall as many as they can, in any order. In subsequent trials, participants are presented with only the non-recalled words from the previous trial. These selective reminding trials continue until the participant either recalls all 12 words on three consecutive trials, or until 12 trials have been completed (Randall and Kerns, 2011). From this neuropsychological episodic memory test, a composite score derived from three sub scores—performance on the last trial, continuous long-term retrieval, and last retrieval—was available. A composite measure, called NP_memory henceforth, was created using the average Z-score from the full sample of the parent study (RANN/CR), as described in detail in (Stern et al., 2021). For interpretability, we re-scaled the NP_memory score to have a mean of 0 and a SD of 1 based on scores of the SOFIA study participants.

### Statistical analysis

All statistical analyses were performed in R (version 4.0.3) using the Rstudio IDE (Version 1.2.5033). Alpha was set at 0.05.

### Characteristics of the sample

Demographic characteristics and the behavioral scores were compared across younger (between 20 and 40 years old) and older participants (over 60 years old). For continuous variables we performed a Welsh’s t-test for samples of unequal variance.

### Linear regressions of behavioral scores on demographic characteristics

For descriptive purposes, we first assessed how individual scores were impacted by sociodemographic characteristics of the cohort. We also used linear regressions to regress the RIF effect, the DF effect and the Suppress effect on age, sex and educational attainment in nested models with and without interaction terms. For all regressions, we used age centered at 50 years old and converted to decades. Educational attainment was centered at 15. All calculations were made using standardized values.

### Principal component analysis

We then used principal component analysis (PCA), including the sub-scores from our 3 memory inhibition tasks described above, to evaluate how the various scores differentially contribute to the principal components. PCA was chosen over exploratory factor analysis because the combination of the limited sample size and the number of variables resulted in excessive collinearity given the low variability, causing the model to fail to converge. For the principal component analysis, we selected only some variables from the entire variable set, in order to be able to compare specifically measurements pertaining to memory inhibition and memory retrieval while limiting redundancy between variables, and uninterpretable intermediate scores. We removed NRP- and RP- from the variable set because those variables are only used to calculate the RIF effect (given by NRP^−^ - RP^−^) but independently the scores are not considered to be reliable measures of either retrieval or inhibition. Indeed, RP- can only be interpreted in light of the baseline score (NRP-). RP+ however, is informative of enhanced encoding, and was therefore kept. In the Suppress task, we similarly selected the Suppress index (accuracy and latency) as well as the score for Valid trials (accuracy and latency) which informs of the baseline familiarity for attended items. The variables used to calculate the Suppress index were removed (Lure and Control trial accuracies and latencies). For the DF task, all variables were kept because we were interested in the question of whether the TBR and TBF scores load together on the same component, or if instead they participate to distinct pools of variability.

The current implementation of the PCA relied on singular value decomposition. To facilitate interpretation of the factor loadings, we additionally performed a direct oblique (Oblimin) eigenvector rotation. To account for measurement scale differences between the variables, we used the standardized (scaled and centered) data matrix. We selected the number of principal components based on Horns’ parallel analysis (Horn, 1965), and retained for interpretation and discussion components with contributions to principal components >0.40, as this is considered the cut off for practical relevance (regardless of sample size) by Stevens (1992), and, according to Hair et al. (1998), p. 112), is the minimum for our sample size (*N* = 200). To explore the reliability of the measures, we computed McDonald’s omega (*ω*).

### Principal component regressions

To determine if the principal components we obtained from the PCA varied with sociodemographic factors, we regressed the principal component scores on age, sex and education using linear models. Additionally, in the subsample with available memory neuropsychological assessment (NP_memory), the principal components were also regressed on NP_memory in addition to age, sex and education.

## Results

### Characteristics of the population

The age class distribution was 59% older (*n* = 104), and 41% younger (*n* = 71) and showed equivalent specific standard deviations (Older: 69.33 ± 5.91; Younger: 30.72 ± 5.4). The self-reported sex at birth composition and educational attainment were balanced in both age classes (including % female in the younger and older age groups, respectively). Participants were asked to provide their sex and were not additionally asked to provide their self-reported gender identity.

Baseline episodic memory retrieval efficiency on the Directed Forgetting task was equivalent between older and younger participants with respective accuracies (proportion correct) on TBR items of 0.82 (± 0.17) and 0.83 (± 0.16). The ‘forget’ instruction given to participants while they held TBF items in working memory led to a decrease in episodic memory retrieval accuracy of 28% in the younger adults (TBF accuracy: 0.55 ± 0.24) vs. only 18% in the older adults (TBF accuracy: 0.64 ± 0.22). This resulted in a significant difference in the DF effect (given by the difference TBR-TBF) between older (0.19 ± 0.21) and younger (0.28 ± 0.26; *t* = 1.9805, *p* = 0.012) participants.

For the Retrieval Induced Forgetting task, accuracy of unpracticed items (NRP- items) was lower (*t* = 3.5039, *p* = 0.001) in the older participants (0.85 ± 0.18) than in the younger participants (0.92 ± 0.08), indicating weaker encoding in the older adults, which was restored to the same level as the young by practice training (RP^+^ items: older, 0.97 ± 0.8; younger, 0.96 ± 0.11, *t* = −1.0806, *p* = 0.282). However, the lower baseline efficiency in the older participants caused the RIF effect (given by the difference between NPR- and RP- accuracies) to be of equivalent magnitude between the two age classes (older: 0.07 ± 0.22; younger: 0.07 ± 0.14; *t* = −1.364, *p* = 0.28).

For the Suppress task, accuracy was not different between older and younger participants across any of the different types of trials (Valid, Lure, Control). Similarly, we found no difference in the memory suppression index (given by the difference between Lure and Control trials). However, despite similar accuracy for the suppression indices between older and younger participants, we found that latency on both the Control and the Lure trials were significantly higher for the older participants relative to the younger participants, and that the associated latency differences between these two types of trials were significantly higher for the older relative to the younger participants as well (latency difference Control – Lure: old, −347.02 ± 265.50; young −232.48 ± 231.34 milliseconds, *t* = 3.399, *p* = <0.001), indicating that maintaining the same accuracy required a higher time cost for older participants. Finally, the latency for Valid trials was also significantly higher for older participants (1567.78 ± 361.90 vs. 1268.44 ± 402.90 in the young; *t* = −5.377, *p* < 0.001). These results are shown in Table 1.

**Table 1:** Description of the sample and behavioral performance (mean, SD) for all task measures.

	Overall (*n* = 175)		Older (*n* = 104)		Younger (*n* = 71)		*p* value
Age	53.66	(19.85)	69.33	(5.91)	30.72	(5.45)	-
Sex = Female (%)	106	(60.60)	59	(56.70)	47	(66.20)	0.218^#^
Race (%)							-
Asian	16	(9)	1	(0.9)	15	(21.1)	
Black or African American	26	(14.8)	17	(16.3)	9	(12.6)	
> one race	10	(5.7)	3	(2.8)	7	(9.8)	
Other	12	(6.8)	4	(3.8)	8	(11.2)	
White	111	(63.4)	79	(75.9)	32	(45)	0.787^#^
Education	0.67	(1.98)	0.63	(2.09)	0.72	(1.83)	-
Directed forgetting							
**TBR**	0.82	(0.16)	0.82	(0.17)	0.83	(0.16)	0.905
**TBF**	0.6	(0.23)	0.64	(0.22)	0.55	(0.24)	0.055
**DF effect**	0.22	(0.23)	0.19	(0.21)	0.28	(0.26)	0.050
Retrieval induced forgetting							
NRP-	0.88	(0.15)	0.85	(0.18)	0.92	(0.08)	0.001
RP−	0.82	(0.19)	0.78	(0.19)	0.89	(0.16)	<0.001
**RP+**	0.97	(0.09)	0.97	(0.08)	0.96	(0.11)	0.282
**RIF effect**	0.06	(0.19)	0.07	(0.22)	0.04	(0.15)	0.174
Suppress							
**Valid (RT)**	1446.33	(405.66)	1567.78	(361.90)	1268.44	(402.91)	<0.001
**Valid**	0.93	(0.09)	0.93	(0.08)	0.92	(0.12)	0.366
Lure (RT)	1763.51	(510.79)	1926.02	(461.72)	1515.24	(484.18)	0.000
Lure	0.87	(0.17)	0.87	(0.17)	0.88	(0.16)	0.427
Control (RT)	1461.81	(419.82)	1582.48	(394.11)	1277.46	(392.20)	<0.001
Control	0.93	(0.13)	0.92	(0.15)	0.95	(0.11)	0.103
**Suppress effect**	−0.06	(0.19)	−0.06	(0.22)	−0.07	(0.14)	0.656
**Suppress effect (RT)**	−300.55	(257.76)	−347.02	(265.50)	−232.48	(231.34)	<0.001

Bolded measures are those included in the Principal Component Analysis. All task values represent accuracy (proportion correct) except where noted. RT, Reaction time, expressed in milliseconds. Education was centered at 15 years. “–“indicates that no test was performed. #For categorical variables we performed one-way ANOVAS comparing the distribution of levels between groups. Educational attainment was treated like a categorical variable because of the limited number of different levels in our sample.

### Impact of sociodemographic characteristics

We evaluated how sociodemographic characteristics –age, sex, and education– of the cohort impacted memory inhibition indices (Table 2). We found that age negatively impacted the DF score, with 2% lower accuracy difference per decade of age (SD = 1%, *p* = 0.05). The Suppress inhibition effect was differently impacted depending on whether we considered accuracy or latency. For latency, we found that the Suppress effect reaction time difference was larger by an average 100.10 milliseconds (± 37.95, *p* = 0.01) in female participants, and this effect was further exacerbated by 50.16 milliseconds (± 19.04, *p* = 0.01) with every additional decade of age, indicating a higher cost in women, and in particular older women. Accuracy score differences were only impacted by educational attainment, with −3% (± 1%, *p* = 0.03) lower values with every additional year of education in male participants only, indicative of a possible positive influence of cognitive reserve on inhibition efficiency. Finally, for the Retrieval Induced Forgetting task, we found a stronger RIF effect with female sex (6% ± 3%, *p* = 0.04).

**Table 2:** Summary of the linear regression showing relation between individual scores on the three tasks and sociodemographic characteristics.

	DF effect	Suppress (RT)	Suppress	RIF effect
Constant	0.24 (0.03)	−241.82 (30.77)	0.04 (0.02)	0.01 (0.02)
	*p* = 0.00***	*p* = 0.00***	*p* = 0.14	*p* = 0.60
Age	−0.02 (0.01)	−9.92 (14.89)	0.003 (0.01)	0.01 (0.01)
	*p* = 0.05*	*p* = 0.51	*p* = 0.66	*p* = 0.11
Education	−0.01 (0.01)	1.22 (9.25)	−0.02 (0.01)	−0.002 (0.01)
	*p* = 0.30	*p* = 0.90	*p* = 0.03*	*p* = 0.74
Age:Sex (F)		−50.16 (19.04)		
		*p* = 0.01**		
Sex (F)	−0.01 (0.03)	−75.72 (38.43)	0.05 (0.03)	0.06 (0.03)
	*p* = 0.75	*p* = 0.06	*p* = 0.09	*p* = 0.04*
Observations	172	172	172	172
*R* ^2^	0.03	0.16	0.05	0.04
Adjusted *R*^2^	0.01	0.14	0.03	0.02
Residual std. error	0.22 (df = 168)	237.22 (df = 167)	0.19 (df = 168)	0.19 (df = 168)
*F* Statistic	1.79 (df = 3; 168)	7.98*** (df = 4; 167)	2.74* (df = 3; 168)	2.16 (df = 3; 168)
Note:				

F, Female.

*p < 0.05; **p < 0.01; ***p < 0.001.

### Principal component analysis

The parallel analysis (Horn, 1965) indicated that 3 components optimally captured the largest amount of variability in our sample, without inflating the noise excessively. We labeled the principal components based on their main variable loadings, respectively: “Instructed Forgetting,” “Memory Facilitation,” and “Uninstructed Forgetting” for components 1 to 3. Table 3 details the individual loadings for each principal component. Component 1 (Instructed Forgetting) was dominated by the DF effect (0.93) and TBF accuracy (−0.94). All other contribution were minimal. Component 2 (Memory Facilitation) received loadings from the practiced items from the Retrieval Induced Forgetting task (RP+; 0.69), the Valid Suppress task trial accuracy (Valid; 0.66) and the to-be-remembered items from the Directed Forgetting task (TBR; 0.70). Component 3 (Uninstructed Forgetting) had dominant contributions from the RIF effect (0.54), the Suppress effect latency difference (−0.83).

**Table 3:** Variable loadings on the principal components after applying a direct oblique rotation (Oblimin).

	Instructed forgetting	Memory facilitation	Uninstructed forgetting	Uniqueness
RIF RP+	−0.07	**0.69**	0.22	0.513
RIF effect	0	0.2	**0.54**	0.704
DF TBF	**−0.94**	0.25	−0.05	0.058
DF effect	**0.93**	0.24	−0.03	0.07
DF TBR	0	**0.7**	−0.1	0.479
Suppress valid (RT)	−0.12	−0.22	0.38	0.773
Suppress valid	0.03	**0.66**	−0.06	0.55
Suppress effect	−0.1	−0.38	**0.5**	0.54
Suppress effect (RT)	−0.06	−0.05	**−0.83**	0.323

All values indicate accuracy, except where noted with RT, reaction time. Contributions equal to 0.4 or higher are bolded. RIF, Retrieval Induced Forgetting; DF, Directed Forgetting; TBF, To be Forgotten; TBR, To be Remembered. Uniqueness indicates the proportion of variability not shared with the other variables.

To test the reliability of the inhibitory measures used in the current study, we computed McDonald’s omega (*ω*) (McDonald, 1999; Hayes and Coutts, 2002). The total ω was 0.73, which provides an acceptable level of support for construct reliability. To further confirm the internal validity of our constructs we compared the principal component scores to sociodemographic factors (Table 4A). We found that Instructed Forgetting was negatively correlated with age (−0.09 standard units ±0.04 per decade of age *p* = 0.02) and Memory Facilitation was higher with more years of educational attainment (0. 10 standard units ±0.04 *p* = 0.01). In the subset of 100 participants with the NP_memory score, the effect of education was weaker when accounting for episodic memory (Table 4B), pointing to a possible interaction between education and NP_memory. NP_memory was mildly predictive of Memory Facilitation scores (0.19 standard units per SD ± 0.12), but with a large variability. Uninstructed Forgetting scores were associated with both age (−0.15 ± 0.04) and with sex (−0.41 standard units ±0.15 in female participants). Finally, neither Instructed Forgetting nor Uninstructed Forgetting were associated with NP_memory.

**Table 4:** Relationship between principal component scores and sociodemographic factors and neuropsychological scores.

		Instructed forgetting	Memory facilitation	Uninstructed forgetting
A	Intercept	0.11 (0.12)	0.02 (0.12)	**0.25 (0.12)**
		*p* = 0.36	*p* = 0.87	***p* = 0.04**
	Age	**−0.09 (0.04)**	−0.01 (0.04)	**−0.15 (0.04)**
		***p* = 0.02**	*p* = 0.74	***p* < 0.001**
	Sex (F)	−0.07 (0.15)	−0.14 (0.15)	**−0.41 (0.15)**
		*p* = 0.66	*p* = 0.37	***p* = 0.01**
	Education	−0.06 (0.04)	**0.10 (0.04)**	**0.08 (0.04)**
		*p* = 0.15	***p* = 0.01**	***p* = 0.04**
B	Intercept	0.04 (0.16)	−0.11 (0.16)	0.20 (0.19)
		*p* = 0.83	*p* = 0.50	*p* = 0.31
	Age	**−0.18 (0.06)**	0.05 (0.06)	**−0.16 (0.07)**
		***p* = 0.01**	*p* = 0.41	***p* = 0.04**
	Sex (F)	−0.07 (0.20)	0.03 (0.20)	**−0.48 (0.24)**
		*p* = 0.74	*p* = 0.88	***p* = 0.05**
	Education	0.02 (0.05)	0.08 (0.05)	0.07 (0.06)
		*p* = 0.61	*p* = 0.11	*p* = 0.25
	NP_Memory	−0.15 (0.12)	0.19 (0.12)	0.0005 (0.14)
		*p* = 0.20	*p* = 0.11	*p* = 1.00

The table shows the regression estimates from 6 different models that evaluate the relationship of the three PC scores with A. Age, Sex (Female) and Education, in the full cohort; and B. In a subset of 100 participants with available neuropsychological episodic memory (NP_Memory) adjusted for age, sex and education. Age and Education were centered at 50 (in decades) and at 15 (in years), respectively. Note the *p* values are not corrected for multiple comparisons. If applying a Bonferroni correction for three tests, the association between sex and Uninstructed Forgetting would not be significant due to the large range of this particular estimate.

## Discussion

In this study, we investigated the relationship between three cognitive tasks commonly used to access memory inhibition. Our primary goal was to determine if the theoretical constructs tested by these tasks aligned with the underlying correlations between performance scores across the three tasks, and to test how each component was impacted by age, sex, and education. Despite an established use of these three tasks in studies of memory inhibition, they had never, to our knowledge, been evaluated together in the same participants. Further, in a subset of 100 of the participants, we additionally evaluated how the principal components related to a neuropsychologic measure of episodic memory.

Across the sub-scores derived from the three tasks that we evaluated, we identified three distinct dimensions that captured (1) Instructed Forgetting (2) Memory Retrieval Facilitation, and (3) Uninstructed Forgetting. The scores from the Suppress effect indices (accuracy and latency differences) and those from the RIF effect (NRp- and Rp- accuracy difference), which both rely on uninstructed memory suppression, loaded together and were independent from the DF effect scores, which loaded on a separate dimension, suggesting that the DF effect may rely on a distinct inhibitory process. This finding is consistent with the existence of two separate memory inhibition processes reflective of the type of instruction provided to the respondent.

The Instructed Forgetting dimension was negatively correlated with age, confirming the differences in TBF and DF effect scores we had seen between younger and older participants (Table 1). This finding supports the idea that the ability to intentionally suppress information while it is held in working memory may decline with age. However, consistent with the finding that the DF score did not correlate with the neuropsychologic episodic memory scores is the idea that during the DF task, a word is first encoded and held in memory until the cue to either forget or to remember it is provided. Interestingly, when comparing accuracy scores of TBR and TBF items between younger and older participants, we found that the “forget” cue did not interfere with TBR accuracy in the older participants, despite larger overall amounts of retained information due to impaired forgetting of the TBF items (TBR 82% and TBF 64% accuracy in the old vs. TBR 83% and TBF 53% accuracy in the young). This suggests that a specific deficit in TBF suppression in the older adults is the cause of age-related differences, rather than a competing memory load (which would have caused lower TBR accuracy). This finding is in contrast with prior work by Salthouse et al. (2006) who found that controlling for TBR scores caused the relation between age and TBF to be null, suggesting that the age-dependent change in DF scores may be driven by a change in how TBR items are processed (Salthouse et al., 2006). But, it is in accord with a meta-analysis by Titz and Verhaeghen (2010), in which the authors proposed that age differences in directed forgetting may be driven by inhibitory mechanisms that operate at the time of encoding and which could explain the important differences observed between item version of the task and list versions like the one used by Salthouse et al. (2006), as well as an imaging study by Ullsperger et al. (2000). Using the DF task, these authors measured event-related-potentials shortly after both cueing and recall and compared the traces of correctly recalled TBR and TBF items. Their analysis revealed the presence of a late right frontal slow wave that persisted after the retrieval of TBF items, which they posited could be reflective of the release of the memory representation of the words from inhibition as, until the moment of the retrieval test, those are items considered irrelevant and are being suppressed. They take this result as evidence that encoding levels, alone, cannot explain the directed forgetting effect.

In our data, Uninstructed Forgetting was influenced by the RIF effect, the Suppress accuracy effect (lower accuracy in lures) and negatively influenced by the Suppress latency effect (additional processing time in lures); the scores were higher in older participants, and in women. This finding may be explained by the individual relationship that the Suppress effect pertaining to latency differences had with age and sex. Indeed, this index also had the highest contribution (−0.83) to the overall component score, and neither the Suppress index of accuracy differences, nor the RIF effect, were affected by age when considered separately (Table 1). This score had a negative loading on the dimension, which could explain the direction of the relationship with age. This suggests that the Uninstructed Forgetting component that we isolated may be dominated by the influence of the extra cognitive cost resulting from ineffectively dropping the lure items. Our findings with the suppress task are largely in agreement with our group’s previous work using the same task, in partially overlapping samples (Eich et al., 2017, 2018; Siegel and Eich, 2021). It is also worth noting that, if we consider accuracy scores alone, our findings are consistent with Collette et al. (2009), who observed age-related differences on two of three tasks that they labeled as indexing “intentional” memory suppression (a short term memory DF task and a long term memory DF task, but not the Hayling task; Collette et al., 2009). Interestingly, Collette and colleagues found no age-related interactions across three tasks that they labeled as tapping ‘unintentional” inhibitory control, including an RIF task, a probe recency task, and a flanker task.

The RIF effect and the accuracy-related Suppress effect were positively correlated with the Suppress latency effect (which indicate longer reaction times for lure items), which indicates that although this portion of the variability is affected by latency, it is not a straight measure of retrieval speed. Instead, it is affected both by the end point accuracy difference at retrieval and by the additional interference cost resulting from partial suppression of irrelevant items (the lures) which too are a measure of suppression. The finding that the forgetting of RP- items in the RIF task, and the lower familiarity of Lure trials in the Suppress task share common variability suggests that a common inhibitory process may be involved when the demands of a task require the manipulation of important and interfering amounts of information in working memory. This could be explained by the fact that some amount of memory load and interference thereof may be necessary to trigger the memory suppression, as was proposed in comparative experiments with and without interference (Rosen and Engle, 1998).

While we found limited literature about the existence of multiple inhibitory processes specific to memory, and the impact aging has on them, the idea of controlled vs. automatic inhibition has been proposed before. Some authors have suggested that inhibition tasks in general may involve executive functions to differing degrees, depending on how consciously the process was initiated (Harnishfeger, 1995; Nigg, 2000; Friedman and Miyake, 2004; Anderson, 2005). And, depending on their degree of involvement of executive functions, various inhibition tasks may be differentially affected by aging (Andrés et al., 2008): the more a task demands executive functions, the more it may be susceptible to age-related impairments due to the decline in executive functions that occurs in aging. Unfortunately, the notions of controlled / automatic inhibition versus instructed / uninstructed forgetting are not entirely similar to one another because the first one relies on the assumed degree of frontal lobe involvement, whereas the second only pertains to how task instructions elicit a voluntary initiation of the process, or not. Indeed, Andrés et al. (2008) considered the RIF task to be among the controlled inhibitory processes, which is consistent with the idea proposed by Anderson and Neely (1996) and Anderson and Hulbert (2021) that the RIF effect is actively engaging cognitive resources, though not consciously. Overall, this body of literature, together with our finding that instructed forgetting and uninstructed forgetting are both impacted by age, suggest that the latter dichotomy could represent an additional distinction within active, controlled inhibitory processes.

An interesting result from our study was the finding of a memory facilitation component that was dissociable from the two memory inhibition components, yet age invariant. This component was comprised of the Valid trials on the Suppress task, the RP+ items from the RIF, and the TBR items from the DF task. All three of these sub scores represent memory fidelity in the absence of conflict: both TBR and Valid items were those items that were tagged, explicitly, to be encoded and maintained for later retrieval; while the RP+ items were encoded incidentally, they were subject to extensive retrieval practice. That the memory facilitation component score was correlated with the episodic memory score derived from classic neuropsychological measures is not surprising, and suggests that our sub scores and component labeling accurately captured episodic memory. What is surprising is that this component showed no age-related effect: episodic memory failure is the hallmark cognitive symptom of cognitive aging, and yet in our data, when this measure was dissociated from inhibitory-related executive control functions, age was not a correlate. The measure did, however, correlate with education, a finding consistent with a large body of literature surrounding the idea of cognitive reserve (Stern, 2009). These results, coupled with the age-related effects on PCs 1 and 3, which both related to different aspects of memory suppression, lend further support to the idea put forth by Hasher and Zacks (2006), that episodic memory, *per se*, is not impaired in aging, or at least is not the first cognitive ability to decline, and instead it is the inhibitory executive component that contaminates many traditional memory tasks that leads to apparent memory failures in older adults.

## Limitations

Our study has several notable limitations. First, our sample may not be representative. This is apparent by virtue of the near ceiling performance across the three tasks, particularly on the sub scores that composed the Memory Facilitation principal component, as well as the relatively small range of educational attainment of the sample. Future studies could employ similar techniques using multiple tasks thought to tap a particular cognitive domain to replicate the results of our study more broadly. Further, our study does not address the question of whether two distinct anatomical systems are enlisted by the two memory suppression processes characterized in this study, or if they work in concert, which would help to shed light on differences in neurobiology that lead to different patterns of performance across the lifespan. Likewise, our study only included one-time point, and thus we cannot assess within individual change across our tasks. Longitudinal follow-up studies measuring individual differences in memory inhibition tasks over time are therefore needed to address the question of whether such inability to efficiently suppress memories is a transient difficulty, or is instead predictive of future cognitive decline. In this context, new research avenues of interest could emerge from the ability to finely characterize age-associated changes in older adults, and to identify possible decliners that will need extra care, or intervention; Although both are sensitive to aging, it is not clear whether instructed vs. uninstructed forgetting processes may be differentially impacted by intervention approaches. Research by Padilla and colleagues suggests that life-style factors (frequent physical exercise vs. sedentary behavior) are associated with differences in memory suppression task efficiency (Padilla et al., 2018). Whether physical activity differentially impacts various executive control systems in older adults has not been evaluated.

## Conclusion

Overall, our results suggest the existence three independent sources of variability in memory suppression: two processes relating to forgetting, and one memory retrieval facilitation process. The two memory inhibition processes are initiated differently by intentional vs. unintentional interaction with memory traces. Our study additionally suggests that the two processes vary independent of one other, as demonstrated by segregated variability of the two corresponding sets of behavioral scores. Finally, we found that while these two processes impacted by aging, supporting the idea that both instructed and uninstructed memory inhibition rely on executive control processes that decline with age, they are independent of episodic memory, which was not found to be impacted by age in our sample. These two types of memory suppression may represent a new dichotomy within controlled executive inhibition mechanisms that pertain to active manipulation of information, which is jeopardized by high cognitive and attentional demands.

## Data availability statement

De-identified data, along with the analytic code (in the R programming language) used in this study is available at https://osf.io/n3r8u/.

## Ethics statement

The studies involving human participants were reviewed and approved by Columbia University Medical Center IRB, Protocol #AAAR0884, and The University of Southern California IRB Protocol #UP-19-00408. The patients/participants provided their written informed consent to participate in this study.

## Author contributions

FC performed statistical analysis, created visualizations, and wrote the manuscript. TE conceived the study, acquired data, analyzed and interpreted data, wrote the manuscript, and acquired funding. All authors contributed to the article and approved the submitted version.

## Funding

This research was supported in part by the National Institute of Health, National Institute on Aging Grants R00AG055684 to TE, and R01AG026158 and R01AG038465 to Yaakov Stern. Any opinions, findings, and conclusions or recommendations expressed in this material are those of the authors and do not necessarily reflect the views of the NIH/NIA. The specific hypothesis tested in this manuscript were not preregistered. All data is available to qualified researchers upon written request.

## Conflict of interest

The authors declare that the research was conducted in the absence of any commercial or financial relationships that could be construed as a potential conflict of interest.

## Publisher’s note

All claims expressed in this article are solely those of the authors and do not necessarily represent those of their affiliated organizations, or those of the publisher, the editors and the reviewers. Any product that may be evaluated in this article, or claim that may be made by its manufacturer, is not guaranteed or endorsed by the publisher.

## Supplementary material

The Supplementary material for this article can be found online at: https://www.frontiersin.org/articles/10.3389/fpsyg.2022.1020915/full#supplementary-material
