# Supplementary material for: Principal component analysis suggests multiple dimensions of memory inhibition that are differentially affected by age
Source: Front Psychol. 2023 Jan 30;13:1020915. doi: 10.3389/fpsyg.2022.1020915 (PMC9941998; doi:10.3389/fpsyg.2022.1020915)
Supplement: Supplementary file 1 [file Data_Sheet_1.PDF]

Appendix

Directed Forgetting

Pencil	Chain	Worm	Mutant	Burglar
Race	Embassy	Mosaic	Genius	Picnic
Plant	Worm	Cricket	Organ	Feather
Chair	Mosaic	Grease	Insect	Heaven
Girl	Cricket	Statue	Belly	Ear
King	Grease	Wrist	Column	Prairie
Obstacle	Statue	Victim	Relic	Circus
World	Wrist	Hunter	Square	Soldier
Macaroni	Victim	Record	Trial	Raisin
Tiger	Hunter	Turtle	Palace	Stove
Sea	Record	Reptile	Expert	Glass
Shower	Turtle	Food	Quarter	Key
Lipstick	Reptile	Cork	Forest	Friend
Cable	Food	Termite	Throat	Entity
School	Cork	Ransom	Steam	Loot
Street	Termite	Purse	Party	Mermaid
Finger	Ransom	Camel	Cabbage	Cousin
Bottle	Purse	Surgeon	Meteor	Twig
Number	Camel	Galaxy	Uniform	Scalp
Movie	Surgeon	Woman	Prophet	Carpet
Mistake	Galaxy	Shadow	Vaccine	Styling
Window	Woman	Studio	Slave	Ghost
Shop	Shadow	Rust	Resin	Company
Language	Studio	Trumpet	Bread	Artist
Message	Rust	Servant	Casino	Garage
Choir	Trumpet	Candy	Emperor	Hair
Accent	Servant	Autumn	Harbor	Exam
Blood	Candy	Beard	Garbage	Tissue
Advisor	Blanket	Meadow	Holiday	Name
Balloon	Dragon	Candle	Monster	Child
Blanket	Chain	Calendar	Frame	Watch
Dragon	Embassy	Match	Mustard	Rubbish
		Soup	Bride	

Retrieval Induced Forgetting

PROFESSION
Banker
Engineer
Cook
Lawyer
Nurse
Teacher
Secretary
Manager
Policeman
Professor
Accountant
Fireman

KITCHEN
Bowl
Mixer
Cup
Fork
Spoon
Pan
Plate
Ladle
Blender
Pot
Whisk
Spatula

EARTH FORMATION
Canyon
Glacier
Boulder
Volcano
River
Hill
Plateau
Island
Cliff
Lake
Ocean
Mountain

SPEECH
Conjunction
Word
Sentence
Verb
Adjective
Noun
Vowel
Syllable
Participle
Pronoun
Adverb
Preposition

DANCE
Modern
Cha Cha
Swing
Tango
Ballet
Waltz
Foxtrot
Square
Merengue
Jazz
Salsa
Tap

VEHICLE
Moped
Skateboard
Jeep
Bus
Truck
Van
Subway
Cab
Scooter
Train
Bike
Taxi

TIME UNIT
Week
Eon
Century
Month
Year
Day
Millennium
Decade
Millisecond
Minute
Second
Hour

AMERICAN CITY
Boston
Houston
Chicago
New York
San Francisco
Los Angeles
Detroit
Philadelphia
Orlando
Seattle
Miami
Washington DC

Suppress Task

MILL
ARTS
PEER
DIVA
BRAN
MASK
MAID
LIMP
MARK
IDEA
BELL
SOAR
BULL
BUZZ
SOUP
HAUL
BLOW
WISE
TAIL
LEAF
MUSH
LICK
GALA
CAKE
CHEF
SAIL
MATE
MEAL
CHAR
LIST
BAKE
TOTE
CAVE
WHIG
MINT
TIRE
CORK
LINK
PORT

HOLE
LEAD
LEFT
MAIL
HEAL
MIND
MINE
NECK
NOTE
PARK
PAST
WISH
CAKE
REAR
RISE
ROAD
ROCK
TOES
BATH
SAFE
SAVE
SEAT
SEED
SELF
GIFT
SIDE
SIZE
JOKE
STEP
TAPE
TOWN
BABY
BAND
BASE
BLUE
CALM
CAMP
CLAY
PEAL
DATE

LADY
FAIR
FAST
FILL
FILM
FINE
FLOW
GAIN
BEET
RIDE
JOIN
KIND
KNOW
LINE
LOAN
MAIN
MEAT
MOOD
MOVE
CART
MASK
PLOT
POST
PULL
GATE
SONG
TASK
TEXT
TOUR
MATE
BOOK
CAST
CITY
CREW
FALL
FIRM
TAIL
CASE
DATA
HEAT
